# Integrated Evolutionary Learning: An Artificial Intelligence Approach to Joint Learning of Features and Hyperparameters for Optimized, Explainable Machine Learning

**DOI:** 10.3389/frai.2022.832530

**Published:** 2022-04-05

**Authors:** Nina de Lacy, Michael J. Ramshaw, J. Nathan Kutz

**Affiliations:** ^1^de Lacy Laboratory, Department of Psychiatry, Huntsman Mental Health Institute, University of Utah, Salt Lake City, UT, United States; ^2^Department of Applied Mathematics, AI Institute in Dynamic Systems, University of Washington, Seattle, WA, United States

**Keywords:** artificial intelligence, machine learning, deep learning, optimization, explainability, feature selection, automated, hyperparameter tuning

## Abstract

Artificial intelligence and machine learning techniques have proved fertile methods for attacking difficult problems in medicine and public health. These techniques have garnered strong interest for the analysis of the large, multi-domain open science datasets that are increasingly available in health research. Discovery science in large datasets is challenging given the unconstrained nature of the learning environment where there may be a large number of potential predictors and appropriate ranges for model hyperparameters are unknown. As well, it is likely that explainability is at a premium in order to engage in future hypothesis generation or analysis. Here, we present a novel method that addresses these challenges by exploiting evolutionary algorithms to optimize machine learning discovery science while exploring a large solution space and minimizing bias. We demonstrate that our approach, called *integrated evolutionary learning* (IEL), provides an automated, adaptive method for jointly learning features and hyperparameters while furnishing explainable models where the original features used to make predictions may be obtained even with artificial neural networks. In IEL the machine learning algorithm of choice is nested inside an evolutionary algorithm which selects features *and* hyperparameters over generations on the basis of an information function to converge on an optimal solution. We apply IEL to three gold standard machine learning algorithms in challenging, heterogenous biobehavioral data: deep learning with artificial neural networks, decision tree-based techniques and baseline linear models. Using our novel IEL approach, artificial neural networks achieved ≥ 95% accuracy, sensitivity and specificity and 45–73% *R*^2^ in classification and substantial gains over default settings. IEL may be applied to a wide range of less- or unconstrained discovery science problems where the practitioner wishes to jointly learn features and hyperparameters in an adaptive, principled manner within the same algorithmic process. This approach offers significant flexibility, enlarges the solution space and mitigates bias that may arise from manual or semi-manual hyperparameter tuning and feature selection and presents the opportunity to select the inner machine learning algorithm based on the results of optimized learning for the problem at hand.

## Introduction

The last decade has seen the rapid adoption and implementation of artificial intelligence and machine learning (AI/ML) algorithms across the engineering, physical, social, and biological sciences. Indeed, new and powerful techniques have allowed for novel applications in science and industry. Perhaps most prominently, the family of methods known as *deep learning* using various types of artificial neural networks (ANN) have made a significant impact given their demonstrable value in both supervised and unsupervised machine learning applications. In biomedical and healthcare research, there has been considerable interest in applying these techniques to discovery science in the large, multi-domain datasets that are increasingly appearing in many fields. Here, practitioners may have available hundreds or thousands of potential predictors with which to construct a model. Moreover, in approaching novel problems, rules-of-thumb or heuristics may not be available for model training parameters. While not limited to biological and biomedical science, this type of less- or unconstrained learning environment tends to be more common in these domains in contrast to physical science or industrial applications. Three challenges commonly impede the formulation of optimized, useful AI/ML models, particularly when the practitioner wishes to investigate high-dimension, heterogenous biomedical data in a less- or unconstrained modeling environment.

Firstly, a shared requirement of the large variety of AI/ML methods is for the practitioner to appropriately parameterize their designs with hyperparameters that optimize learning (Claesen, 2015). These settings can have dramatic effects on results and performance. The problem may be usefully considered as two related sub-issues: the optimization of hyperparameters that control learning and determination of when model training may be terminated. Currently, hyperparameters are often manually tuned and the number of model training iterations is empirically selected and frequently small. For instance, a recent large survey of practitioners found that the majority pursued ≤ 50 model fits in computational experiments (Bouthillier and Varoquaux, 2020). Reliance on manual hyperparameter search or rules-of-thumb (Hinton, 2012) can affect reproducibility, result in sub-optimal solutions or become impractical when the number or range of the hyperparameters is large. In particular, deep learning models are somewhat notorious for being difficult to “tune,” or optimize. Moreover, in early-stage or discovery science, rules-of-thumb may be unavailable because the problem is novel. There is increasing interest in developing automated methods to perform hyperparameter tuning and evidence that these outperform manual approaches (Bergstra. J., 2011; Bergstra and Bengio, 2012). Ideally, such an automated method would select from a wide range of potential hyperparameter settings and converge upon an optimized solution in a principled manner.

Secondly, research often takes as its substrate for AI/ML high-dimension datasets with many potential predictors that may also be of heterogenous types. In particular, the collection and release of multi-domain open science datasets for discovery is a strong trend in biomedicine. While the ability of AI/ML techniques to simultaneously analyze large predictor sets is a strength over more conventional statistical methods, the problem of feature selection (the extraction of a reduced set of features that best represent the analytic problem) becomes intense in these less-constrained learning contexts. Deep learning does not require explicit feature selection and in this sense can be a boon in saving the practitioner the necessity of performing explicit feature selection. However, dimensionality reduction effected through feature selection remains an important step in the analysis pipeline, especially in exploratory analyses or discovery science in high-dimension datasets. It reduces overfitting and renders the training process more computationally efficient. When manual or semi-manual feature selection is used in machine learning, model bias or a solution space that is cramped may result. Moreover, manual feature selection relies heavily on expert domain knowledge that may not be available when the problem or data are novel.

Finally, explainability is a priority in biomedical and/or translational applications. Conventionally, ANNs learn by constructing machine-generated intermediate features that are not interpretable by humans. However, in certain applications such as healthcare this can be a drawback. For instance, the broad aim of precision medicine is to construct disease models that predict the risk of an individual patient for a disease outcome and/or predict their personal response to a specific intervention. In this case, we will very likely want to know which original, human-interpretable features act as predictors in the deep learning model and their relative importance, since these will likely be the targets of interventions and treatments. Further, knowledge about which original features are important can support hypothesis-formation for future work, the discovery of biological mechanisms and the formation of future experimental samples.

Motivated by these challenges to modeling in less- or unconstrained learning environments, we developed a novel AI method that exploits the principles of evolutionary algorithms to produce convergent, optimized solutions. Evolutionary learning algorithms are metaheuristics inspired by biological evolution with a rich history in computational intelligence across many scientific fields (Vikhar, 2016). Here, we present our approach of integrated evolutionary learning (IEL), which provides an automated AI strategy for jointly learning features and hyperparameters while also furnishing explainable machine learning models where the original features used to make predictions may be obtained and ranked in order of importance. IEL is an example of narrow or applied AI, where an adaptive algorithm functions autonomously in response to newly encountered data. A machine learning algorithm of choice is nested inside IEL which acts upon the ML model to select features and hyperparameters over many learning generations based on an information theoretic fitness function to converge on an optimal solution. In short, IEL is an AI method which jointly perform feature selection and leverages evolutionary learning optimized over the hyperparameters in order to achieve the best performance i.e., the hyperparameter tuning is optimized for performance. It was designed to address the problem of discovery science in large multi-domain datasets and differs from other automated or semi-automated approaches to either feature selection or hyperparameter tuning since it selects both features and hyperparameters within the same adaptive learning process. Thus it jointly trains and learns features and hyperparameters. To demonstrate IEL's potential to optimize AI/ML for translational applications in health science in complex multi-domain data, we present a challenging use case in optimizing classification in bio-behavioral “big” data. Comparison with conventional training and testing of machine learning algorithms using default hyperparameter settings is also performed to demonstrate the performance improvement accruing from the use of IEL.

## Materials and Methods

### Data

Our experiments use data from the ongoing Healthy Brain Network (HBN) study by the Child Mind Institute (Alexander et al., 2017). The HBN initiative collects multi-domain data from youth with at least one behavioral concern aged 5–21 years old in the New York City area comprising behavioral, social, cultural, economic, biological and neural data. We selected participants with at least one complete resting-state functional MRI (fMRI) scan (365 volumes), available phenotypic data to Release 8 and complete data for the predictive target measures of interest (Alexander et al., 2017). Demographic features of the total sample are presented in Table 1.

**Table 1:** Demographic and cognitive characteristics of participant sample.

**Characteristic**	**Range**	**Mean**	**Median**
Age	5.1 to 21.5	10.8	9.9
FSIQ	42 to 147	98.2	100
Autism traits	0 to 47	7.2	4
Handedness	(−100) to (+100)	59.4	77.8
Dimensional change	0 to 100	34.2	25.0
Inhibitory control	0 to 99	26.2	19.0
Working memory	0 to 100	41.3	37.0
Pattern recognition	0 to 100	39.0	32.0

*Characteristics of 1,120 participants in the study are shown. The sample contained 729 male youth and 391 female youth*.

*FSIQ = full scale intelligence quotient, assessed with the Wechsler Intelligence Scale (WISC 5). Handedness was determined with the Edinburgh Handedness Inventory, where a score of −100 represents maximal left dominance and +100 maximal right dominance. For cognitive measures, dimensional change was assessed with a card sort task, inhibitory control with a flanker task and working memory with a list sorting task*.

The participant sample was randomly split with ~70% used for training and testing and ~30% reserved as unseen validation data with the data preparation pipeline applied separately. In the present study, “testing” and “test set” refers to the set of examples used to jointly learn features and hyperparameter settings. The terms “validation set” and “validation” refer to the set of examples used only once to assess the performance (i.e., generalization) of the fully specified classifiers or regression models. The HBN study was approved by the Chesapeake Institutional Review Board. The present study was deemed not human subjects research by the University of Washington Review Board and the University of Utah Review Board.

### Feature and Target Selection and Preparation

#### Bio-Psycho-Social Feature Selection and Preparation

**Figure 1:**
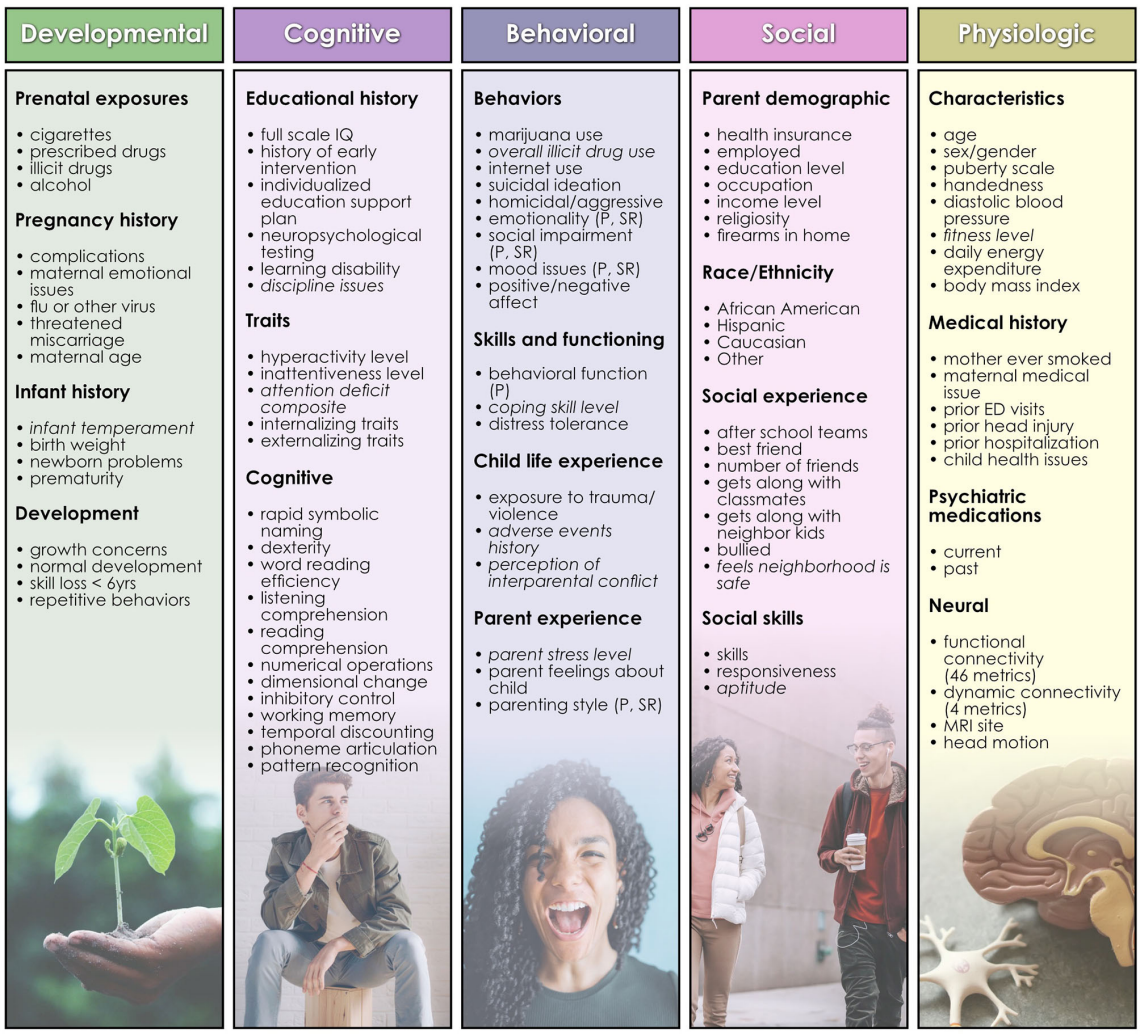
Features used in predictive analytics. Developmental, cognitive, behavioral, social, and physiologic features used as predictors in analytic experiments are shown, organized by type. Terms in *italics* are those for which feature agglomeration was applied to generate a summative metric. A list of the names of underlying assessments used to generate each feature and further descriptions of features may be inspected in Supplementary Table 1.

HBN collects data from participants in 4 study visits of 3 h with a standardized protocol: http://fcon_1000.projects.nitrc.org/indi/cmi_healthy_brain_network/index.html. For continuous measures, we selected the available summary or total metric. For 11 instruments (Figure 1) no such metric was available and we computed a summary measure by applying feature agglomeration to recursively merge individual items and generate a single continuous measure. Features with >40% missing values were discarded, and continuous variables trimmed to mean ± 3 standard deviations to remove outliers. Missing values for the remaining variables were imputed using non-negative matrix factorization. No Matches Found All features were then scaled using scikit-learn's MinMaxScaler (Lee and Seung, 1999; Jain et al., 2013). Features with skewed distributions were transformed with scikit-learn's Quantile and Power transforms and the post-transform feature most closely resembling a normal distribution was selected for inclusion in the predictor set. 110 bio-psycho-social features were included in the study (Figure 1) for predictions of problem behaviors (CBCL) and 109 features for prediction of life function (WHODAS) and autism (ASSQ) since the WHODAS and ASSQ were, respectively, removed from the feature set in experiments where these metrics served as the target of prediction.

#### Neural Connectivity Features

We computed gold standard brain functional connectivity measures from functional MRI (fMRI). HBN acquires multiband 3T resting-state, eyes open fMRI comprising 365 volumes at 2 sites. After removing the first 10 volumes to allow for scanner equilibration, each participant's scan was realigned, coregistered, normalized and smoothed at 6 mm full width at half maximum using standard algorithms in SPM12 (https://www.fil.ion.ucl.ac.uk/spm/software/spm12/). These pre-processed scans were then submitted to quality control by computing correlation with a group mask and 21 participants with <90% correlation with this group mask were eliminated. Head motion was computed for each participant with the DVARS (Christodoulou et al., 2013; Power et al., 2014) metric. We then used an established pipeline to perform group spatial independent component analysis to extract a whole brain parcellation scheme representing 15 functional components (Allen et al., 2011) with the widely used Group ICA of fMRI Toolbox (GIFT) (Calhoun et al., 2001; Calhoun and Adali, 2012). Spatial ICA is a standard method to estimate biological gray matter neural networks from fMRI signals. Components estimated by ICA were sorted into gray-matter intrinsic functional networks vs. artifactual noise components with a combination of expert visual inspection by NdL and the quantitative metrics of fractional amplitude of low frequency fluctuations and dynamic range (Allen et al., 2011). Components with poor overlap with cerebral gray matter or low spectral metrics were discarded and we retained a set of 10 functional intrinsic neural networks (IN). We constructed a spatial map for each IN following an established GIFT pipeline (Allen et al., 2011). To determine functional connectivity strength among INs, we computed Pearson correlations among each possible pair of spatial maps.

An additional set of dynamic connectivity metrics was computed by delineating stable dynamic whole-brain connectivity states from the fMRI ICA timecourses and applying the temporal ICA (tICA) clustering algorithm to connectivity windows using an established sliding window method (Sakoglu et al., 2010; Allen et al., 2014). This approach aims to construct metrics that describe the fluidity and range with which participants traverse brain states. We applied the tICA algorithm to the windowed covariance matrices using the city method to compute the connectivity patterns (CPs) and discretized the time-varying, additive contributions made by CPs to each observed windowed covariance matrix. A 4-dimensional weight vector was obtained representing the contribution of each CP to each matrix by regressing the functional connectivity estimate onto the tICA cluster centroid. Real-valued weights accruing from this computation were then replaced by a value in ± (Bergstra and Bengio, 2012; Hinton, 2012; Claesen, 2015; Bouthillier and Varoquaux, 2020) according to the signed quartile into which each weight fell. The resulting discretized vectors are termed “meta-states.” Four metrics dynamism were computed for these meta-states. Two metrics describe the fluidity with which subjects traverse the meta-state space: the number of distinct meta-states passed through by each individual and the number of times each subject switches between meta-states. The remaining two metrics describe the high-dimension dynamic range achieved by subjects: the maximal L^1^ span achieved between occupied meta-states, and the total distance “traveled” by an individual through the state space (sum of all L^1^ distances). A total of 50 neural features were included in the study (Figure 1).

Nuisance regressors of scanner site, DVARS statistic and 6 realignment parameters and their 6 first derivatives for each participant were regressed from all connectivity models using the general linear model prior to computing. Scanner site and head motion (DVARS) were retained as features in the predictive analytics to assess for any residual effects.

#### Predictive Targets

Well-validated, widely-used gold standard behavioral measures were selected from the HBN dataset for use as predictive targets. Since we selected participants with complete predictive target data for each experiment, numbers of participants used for training/testing and validation varied slightly among experiments. The World Health Organization Disability Schedule (WHODAS 2.0) was used as a metric of daily life function. The WHODAS asks about difficulties due to illnesses such as mental or emotional problems and surveys communication, mobility, self-care, relationship function and participation in work and social activities. Predictive experiments involving the WHODAS used input matrices of size 766 × 159 (subjects × features for train/test) and 326 × 159 (validation). The Autism Spectrum Screening Questionnaire (ASSQ) is measures traits and behaviors related to autism with answers solicited from parents. It is considered to be a useful screen of autism-related behaviors in “high functioning” youth. Predictive experiments involving the ASSQ, where the WHODAS was included as a feature, used input matrices of size 766 × 160 (train/test) and 326 × 160 (validation). The Child Behavior Checklist (CBCL) is used to detect behavioral and emotional problems in children and adolescents. It surveys anxious, depressed, somatic, social, thought, attention, rule-breaking and aggressive behaviors. Predictive experiments involving the CBCL used input matrices of size 722 × 160 (train/test) and 310 × 160 (validation), since fewer subjects with complete CBCL data were available. For each of these continuous measures we used the total score for regression analyses. To convert these scores into cases for classifications, a threshold was determined from the continuous score distribution for each illness target that divided participants into a group with no appreciable symptoms and another with a range of symptom severity. The former group were considered not a case where the latter was deemed a case. Each sample was balanced as far as possible using synthetic oversampling with the SMOTEENN algorithm.

### Predictive Analytics

**Figure 2:**
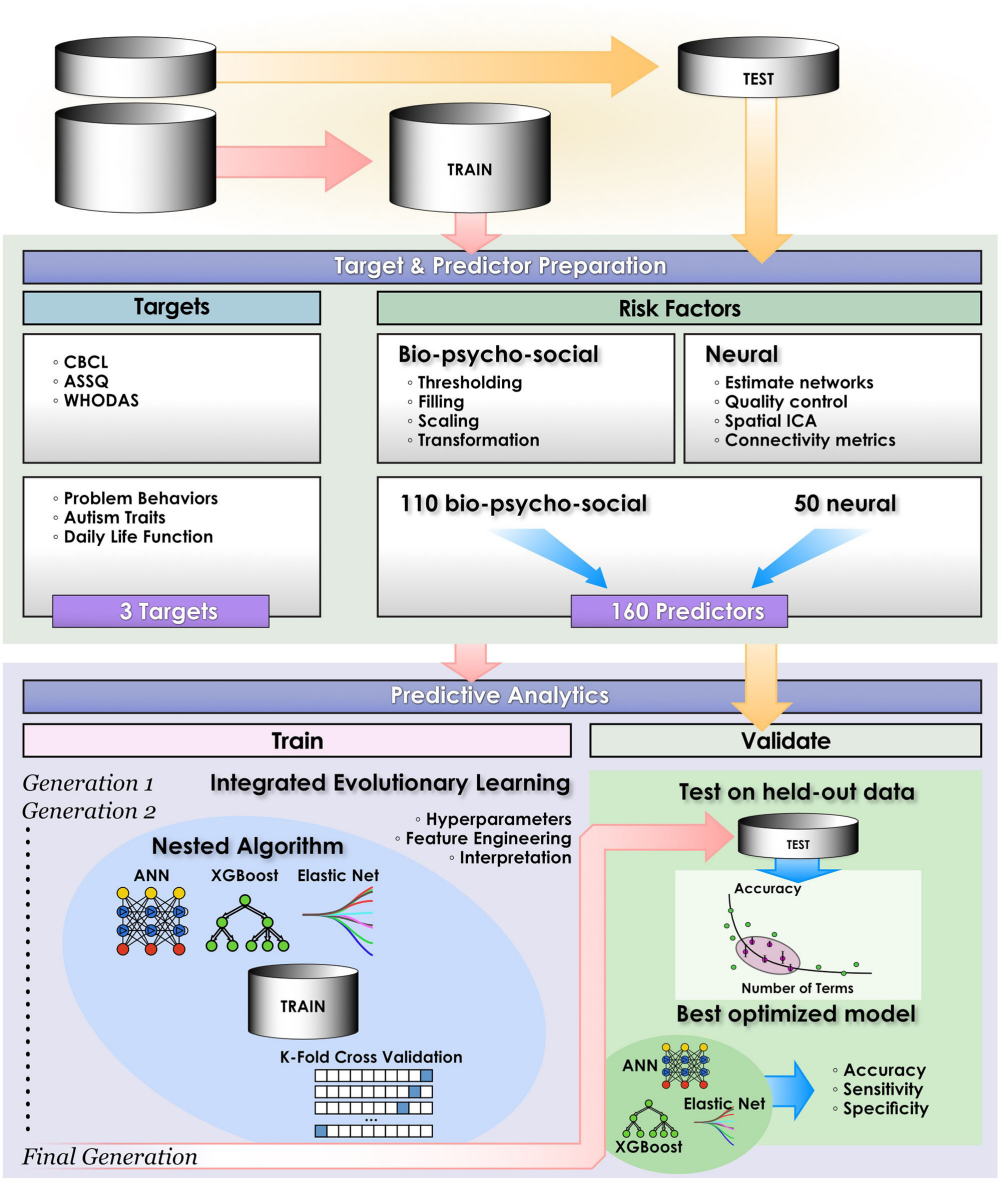
Computational pipeline. The study computational pipeline is shown as detailed in materials and methods for classification experiments. In regression experiments the pipeline was similar, with the exceptions that only deep learning with artificial neural networks was performed and performance statistics of Mean Squared Error, Explained Variance and *R*^2^ were computed.

We compared the ability of three leading ML techniques to predict daily life function (WHODAS) and autism traits (ASSQ) in classification approaches optimized with IEL (Figure 2): deep learning with ANNs, gradient-boosted decision tree-based learning and a benchmark linear model. To demonstrate the ability of IEL to perform in regression, we also performed regression-based prediction with deep learning for daily life function (WHODAS) and problem behaviors (CBCL). To optimize learning performance, each algorithm was applied within IEL, our evolutionary algorithm framework with k-fold cross validation in custom Python code. Training and testing of each individual model within every IEL learning generation for all ML algorithms was performed with cross-validation as detailed below (section Cross-validation). After training and testing over many generations, a small set of optimized models is identified and final validation performed on the held-out dataset to determine how well this small subset of optimized models generalizes to unseen data (section Validation). In addition, each predictive experiment was repeated for each target and ML method without IEL using the default hyperparameter settings for each ML algorithm.

Motivated by the knowledge that not all researchers have access to multi-GPU environments, each deep learning experiment in the present study was performed on one GPU without the use of parallel computing. The code for predictive algorithms with IEL may be accessed in our laboratory GitHub (https://github.com/delacylab/integrated_evolutionary_learning). Pseudocode is also provided as Supplementary Table 2.

#### Deep Learning With Artificial Neural Networks

We trained and tested ANNs using the Adam algorithm with 3 layers, 300 neurons per layer, early stopping (patience = 3, metric = validation loss) and the Relu activation function. The last output layer contained a conventional softmax function for classification analyses. The Adam algorithm was selected based on its established computational efficiency and suitability for problems with a large number of parameters like our study (Kingma and Adam, 2017). Learning parameters (Table 2) were tuned with IEL and the relative importance of each risk factor determined by embedding eli5 (https://eli5.readthedocs.io/en/latest/index.html), an established permutation algorithm, within the IEL algorithm (Breiman, 2001). Hyperparameters that we tuned in the present experiments may be viewed in Table 2 and corollary default hyperparameter settings in **Table 7**. Deep learning models were encoded with TensorFlow embedded in custom Python code.

**Table 2:** Hyperparameters tuned via integrated evolutionary learning.

**Algorithm type and hyperparameters**	**Range**	**Mutation shift**
**Artificial neural network**		
Learning rate	0.00001–0.01	0.0001
Beta 1	0.9–0.999	0.001
Beta 2	0.9–0.999	0.001
**XGBoost (tree-based)**		
Maximum tree depth	2–10	1
Node partition threshold (gamma)	0–0.00001	0.0000001
L1 penalty (alpha)	0.1–0.9	0.001
**ElasticNet (linear)**		
L1 penalty	0–1	0.01
L2 penalty	0–1	0.01

*For each of the machine learning techniques employed in the study (artificial neural networks, XGBoost and ElasticNet), model hyperparameters were tuned within Integrated Evolutionary Learning to optimize model selection. The maximum range of possible hyperparameter values (Range) for each technique is displayed. For models which underwent mutation, the value of a single mutation shift is shown (Mutation shift)*.

#### Gradient-Boosted Tree-Based Learning

We trained and tested tree-based models to predict mental illness cases with the XGBoost algorithm using the gbtree booster (Friedman, 2001; Chen and XGBoost, 2016). This is an ensemble-based method that generates a multitude of decision trees that “vote” on a composite prediction. It is accurate (Fernandez-Delgado et al., 2014), resistant to over-fitting when properly tuned (Kleinberg, 1996) and uses model residuals (actual–predicted values) to penalize leaves that do not improve predictions, reducing bias as well as variance. Empirically, gradient-boosted techniques have been highly successful (Harasymiv, 2015). Hyperparameters that we tuned in the present experiments may be viewed in Table 2 and corollary default hyperparameter settings in **Table 7**. The importance of each feature was computed within XGBoost, encoded with the Scikit-Learn wrapper in custom Python code.

#### Linear Classification

We trained and tested linear models to classify mental illness cases with the ElasticNet. This regularization method linearly combines the L1 penalty of the LASSO (least absolute shrinkage and selection operator) and the L2 penalty of the Ridge method. It produces superior results in real world and simulated data, particularly to the use of LASSO alone (Zou Ha, 2005). The L1 and L2 parameters were tuned using IEL (Table 2). The relative importance of each risk factor was determined by computing its linear coefficient (beta). Hyperparameters that we tuned in the present experiments may be viewed in Table 2 and corollary default hyperparameter settings in **Table 7**. We encoded the ElasticNet model using Scikit-learn algorithm embedded within custom Python code.

#### Cross Validation

For each of the three ML techniques every one of the individual models throughout each IEL learning generation were fit using stratified *k*-fold cross validation for classification. Since the number of features for each model fit could differ within IEL, *k* (the number of splits) was set as the nearest integer above [sample size/number of features]. Cross validation was implemented for classification analyses with the scikit-learn StratifiedKFold function. For regression analyses, cross validation was implemented with the scikit-learn Kfold function. Cross-validation was similarly incorporated in the comparative experiments using default hyperparameter settings.

### Integrated Evolutionary Learning for Machine Learning Model Optimization

**Figure 3:**
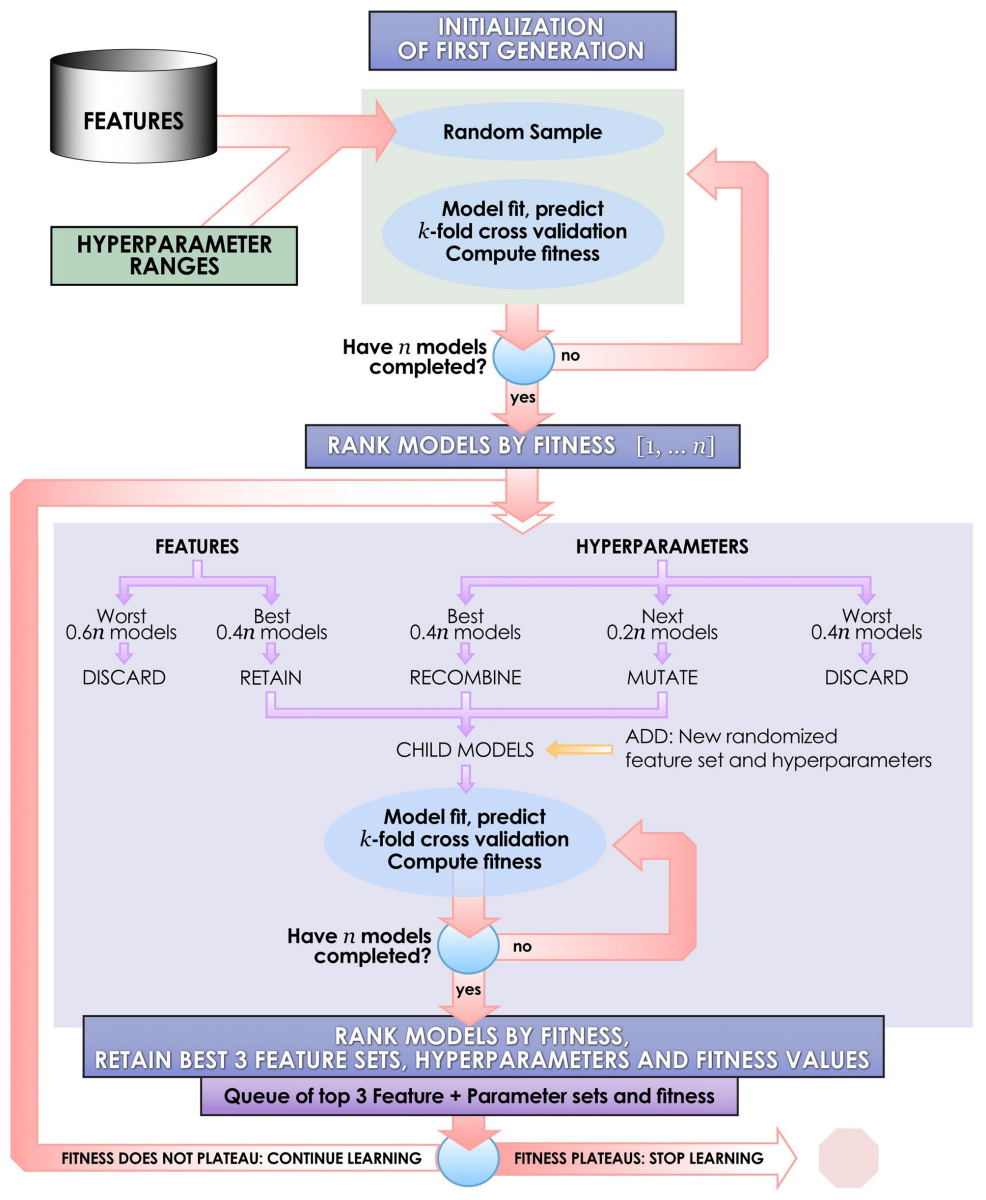
Schematic of the IEL learning process. IEL is an example of a narrow AI algorithm that acts upon a machine learning technique, designed for discovery science in large datasets. IEL is used to jointly learn features and hyperparameter settings for machine learning models in an integrated, adaptive manner over successive learning generations in response to new inputs on the basis of an information theoretic fitness function.

Each ML algorithm was implemented within IEL to optimize model selection and performance (Figure 2). IEL jointly learns features and hyperparameter values over successive learning generations in an integrated manner based on improvements in an information theoretic fitness function. In the present paper, we utilized the Bayes Information Criterion (BIC) as a fitness function to continuously select higher-performing models and discard underperforming solutions but other information theoretic measures such as Kullback-Liebler Divergence could be substituted. For each machine learning algorithm, a first generation of *n* models (in the present experiments *n* = 100) is initialized with “chromosomes” consisting of hyperparameter values selected randomly from a range (Table 2) and 1–50 features selected randomly from the total possible set of ~160 features. The feature set and hyperameters of each of the *n* models is therefore random and different. After training these initial *n* models, the BIC is computed for each of the *n* solutions. Hyperparameter values and features were subsequently recombined, mutated or eliminated over successive generations. Figure 3 shows a schematic of how IEL performs evolutionary selection of features and hyperparameter values.

**Figure 4:**
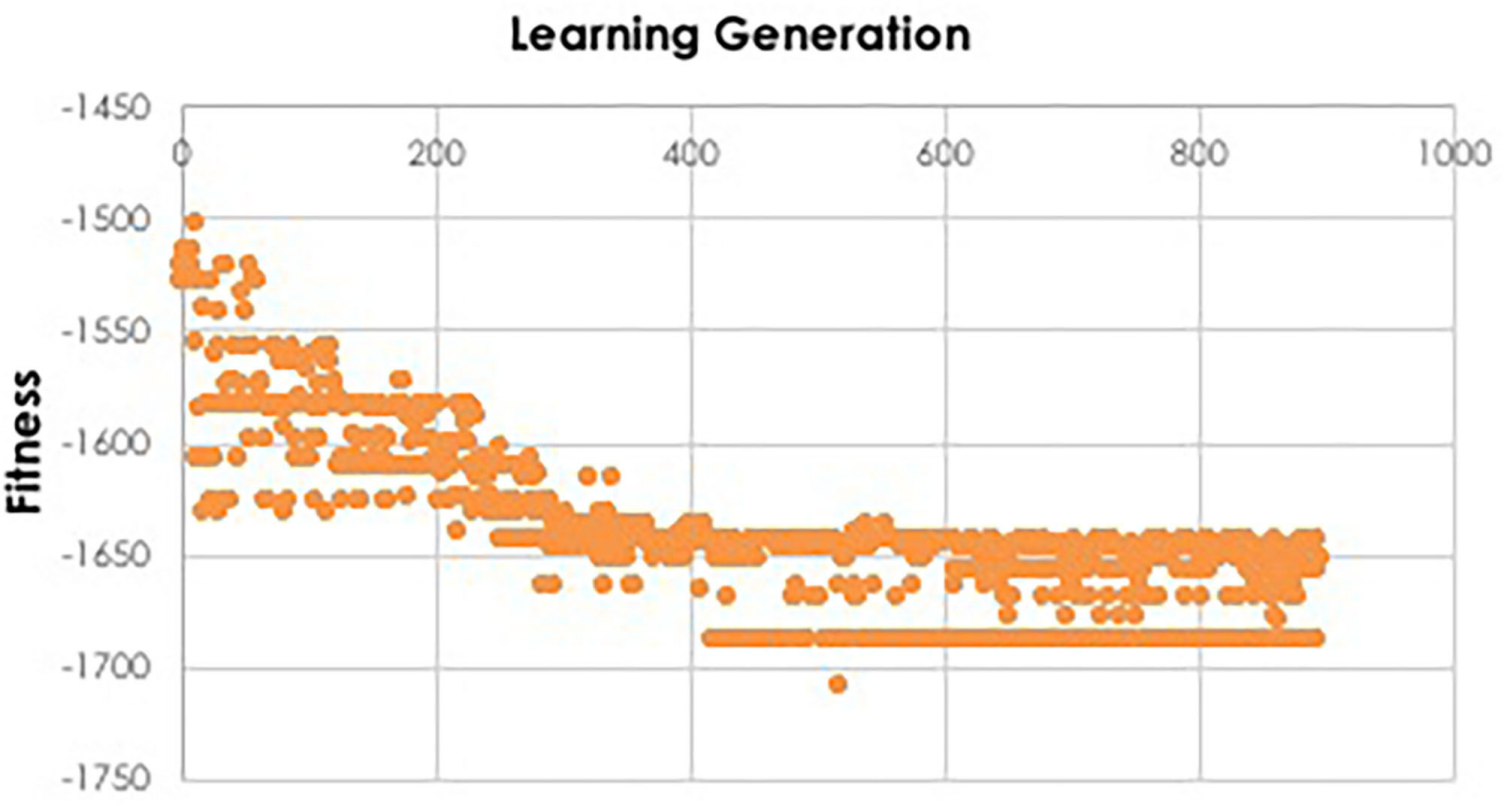
Fitness function during IEL learning. During model training, the fitness function is used by IEL to select best-performing models for recombination and mutation. Initial learning may be terminated when the BIC plateaus, as shown here for training the XGBoost model to predict autism which plateaus after ~130 learning generations or ~400 models, since 3 best-performing models are retained per generation.

In recombination, “parent” hyperparameters of each type are averaged to form “children.” After computing the BIC for the first learning generation, the 0.4*n* best-performing models (here, 40 models) are recombined. For example, in the ANN models, the learning rate of the best and second-best performing models was averaged after a pivot point at the midpoint to establish a new learning rate for the first ‘child' model of the subsequent IEL generation. Thus, hyperparameters of the 0.4*n* = 40 best-performing models of the first generation are recombined to form new hyperparameter values for 0.2*n* = 20 new child models in the next IEL generation. In mutation, hyperparameter settings are shifted. 0.2*n* = 20 best models based on BIC values were mutated to produce the same number of child models by shifting the requisite hyperparameter by the mutation shift value (Table 2). For example, the learning rate for an ANN model was incremented or decremented by 0.0001. The remaining 0.4*n* = 40 lowest-performing models based on the BIC were discarded. The next generation of models was formed by adding 0.6*n* = 60 new models with randomized settings and adding these to the 0.4*n* = 40 child models formed via recombination and mutation for a full complement of *n* models moving forward in the IEL learning process. Thereafter, an automated process continues to recombine, mutate and discard *n* models per generation based on the values of the BIC as new data is encountered by IEL until the fitness function plateaues (Figures 3, 4). The fitness, features and hyperparameters of the 3 best performing models per generation are retained in a queue which is monitored for plateauing in the fitness function i.e., the convergence condition. Excepting the ElasticNet model (which has naturally bounded hyperparameter intervals in [0–1]), possible values for each hyperparameter were generously set to allow for broad exploration of the potential solution set.

As described above, features are jointly learned with hyperparameters during each IEL learning generation. In the initial learning generation, a random number of features in the range [1–50] is set for each of the *n* = 100 models. Here, predictors are randomly sampled from the set of ~160 possible features to create feature sets for each model. After computing the BIC for this first generation of models, feature sets from the best-performing 0.4*n* = 40 models are carried forward to serve as potential predictors in the child models. For example, feature sets used by the best-performing 0.4*n* = 40 ANN parent models were retained and served as potential predictors in the 0.4*n* = 40 ANN child models with hyperparameter settings derived from recombination and mutation. Feature sets for the worst-performing 0.6*n* = 60 models are discarded. Integrated with the hyperparameter tuning described above, the process was repeated for succeeding generations until the BIC plateaued as revealed in the queue of fitness values.

**Figure 5:**
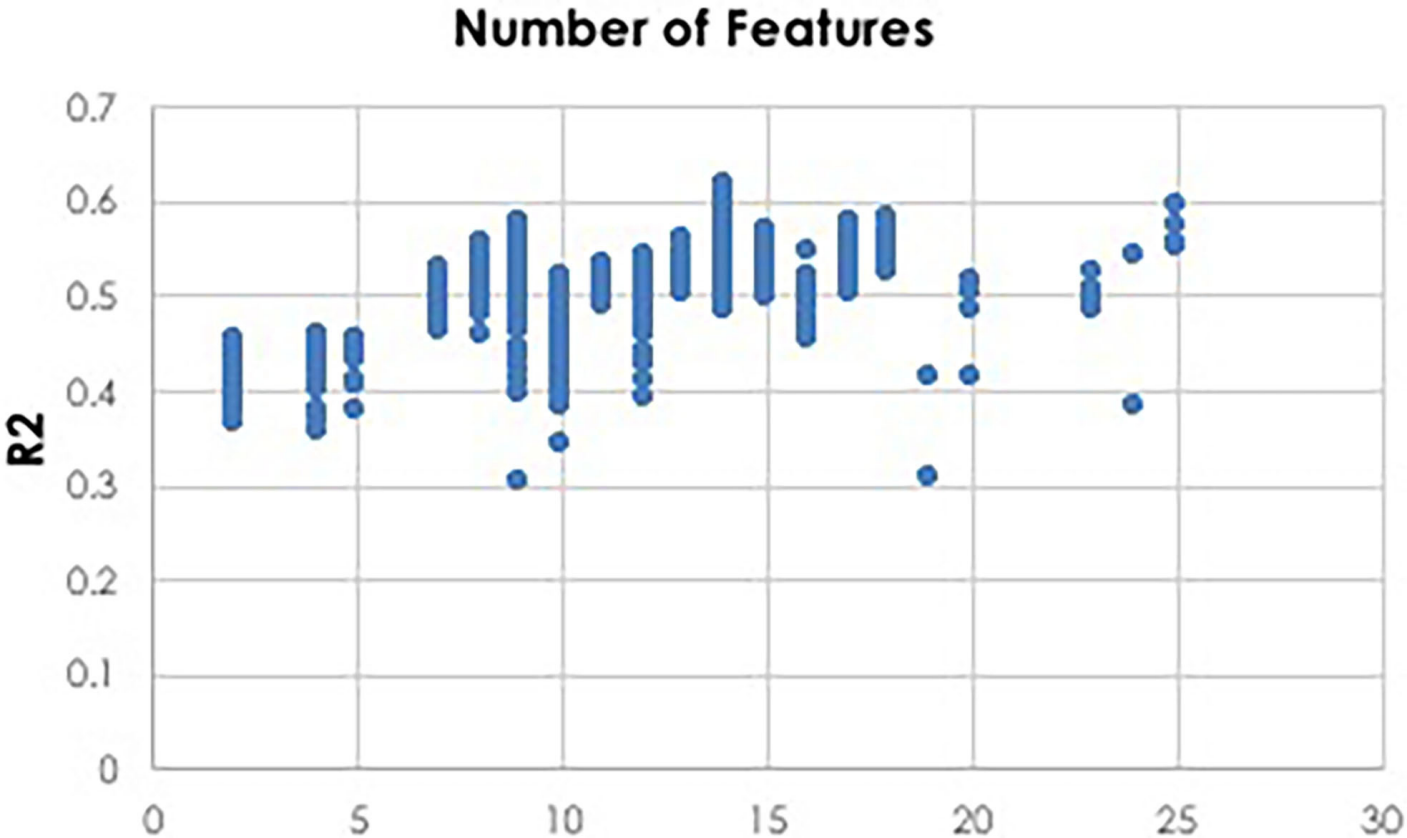
Thresholding for warm start learning. Prior to beginning the recursive training portion of learning, a thresholding process is used to implement feature selection for a warm start to final learning. Here we show an example of deep learning with artificial neural networks to predict CBCL scores using regression. Plotting the *R*^2^ metric against the number of features reveals that 14 features is optimal. A threshold value is then identified which constrains the available feature set for final recursive learning after the warm start to the 14 most important features identified during initial learning prior to the warm start.

To facilitate computationally efficient modeling, IEL implements recursive learning. After training models until the BIC plateaues (Figure 4), we determine the elbow of a performance metric plotted vs. number of features. In the present experiments, accuracy was used for the classifications and *R*^2^ for the regressions, but other metrics such as error or precision may be substituted as the practitioner chooses. The total number of features available after the warm start is constrained to that subset of features, thresholded by their importance, that corresponds to the elbow (Figure 5). For example, the elbow for regression prediction of the CBCL may be identified at 14 features, which corresponds to an importance threshold of 20.0. After the warm start, learning proceeds by constraining features available for learning at increasing thresholds in [warm start feature importance + [0–4] standard deviations]. In addition, we reduce the number of models per generation to 0.5*n* = 50 with 0.2*n* = 20 models recombined and 0.1 = 10 models mutated. Otherwise, after restarting the training process at the warm start threshold ranges an initial generation of models was randomly initialized and training completed using the same principles as detailed above (Figure 3).

### Validating Results on Held-Out Test Data

After training and testing was completed for each model in each generation, the best performing *n* = 100 optimized models were applied to the reserved unseen validation set. We selected statistical tests that are commonly accepted for ML and available across all algorithm types used in this study including deep learning with ANNs. For the classification experiments, performance metrics of accuracy, precision (specificity or positive predictive value) and recall (sensitivity or negative predictive value) were determined. For regression experiments, mean squared error, explained variance and *R*^2^ were computed. Information theoretic measures are another class of metrics that are available for both artificial neural networks and other machine learning algorithm types and we also computed and report the Bayes Information Criterion. For each experiment the single best performing, optimized model was selected based on these performance metrics. Of note, validation testing did not use synthetic oversampling but was performed on native data.

## Results

### Deep Learning With IEL Generalized Most Robustly to Unseen Validation Data

Deep learning with artificial neural networks robustly classified individual life function and autism achieving ≥ 95% accuracy, precision and recall (Table 3) after optimization with IEL. Similarly, when gradient-boosted decision tree learning was optimized with IEL, accuracy and recall of over 99% was realized, though precision was only ~55–60%. In the baseline linear ElasticNet models, performance was also relatively good given that comparison among the methods suggests that this complex biobehavioral data is better modeled with the non-linear deep learning and tree-based algorithms. The linear models offered comparable precision to XGBoost but did not achieve the very high levels of accuracy and recall seen using other techniques.

**Table 3:** Results of classification using 3 algorithm types optimized with IEL vs. default settings.

**Target**	**Accuracy %**	**Precision (Sensitivity) %**	**Recall (Specificity) %**	**Bayes Information Criterion**
**(a)**				
Life function (WHODAS)	96.7	94.6	96.8	−1108.1
*With default settings*	68.4	59.7	70.6	−266.7
Autism (ASSQ)	98.8	98.2	97.9	−1315.6
*With default settings*	74.5	64.6	75.8	−224.7
**(b)**				
Life function (WHODAS)	99.1	60.7	100.0	−1516.8
*With default settings*	68.7	57.0	78.1	−216.4
Autism (ASSQ)	99.7	55.2	99.5	−1869.2
*With default settings*	70.2	50.3	75.1	−289.1
**(c)**				
Life function (WHODAS)	74.5	56.9	85.6	−440.2
*With default settings*	65.1	56.9	68.2	43.3
Autism (ASSQ)	80.1	51.5	90.1	−504.2
*With default settings*	67.2	49.9	65.2	15.2

*The relative classification performance of (a) Deep learning with artificial neural networks; (b) Gradient-boosted tree-based learning and (c) Linear model with ElasticNet optimized with Integrated Evolutionary Learning is shown. Each result is directly compared with performance without IEL using default settings for hyperparameters. Performance metrics for the best-performing model for each algorithm type validated on held-out data to assess the generalizability of the models are shown*.

We also performed regression-based prediction for life function and problem behaviors where the targets were continuous rather than discretized measures. Here, IEL again performed well when optimizing machine learning predictions of life function and problem behaviors, with 46 and 73% explained variance and *R*^2^ respectively (Table 4). Prediction of problem behaviors using the CBCL was more robust than that of life function using the WHODAS. The former is noted to offer a smoother continuous scale than the latter.

**Table 4:** Results of regression prediction using deep learning optimized with IEL.

**Target**	**Mean squared error**	**Explained variance %**	***R* ^2^**	**Bayes Information Criterion**
Life function (WHODAS)	135.1	46.2	45.6	−672.8
*With default settings*	167.2	0.3	0.3	1988.0
Problem behaviors (CBCL)	34.9	72.6	72.6	1,131
*With default settings*	477.5	−0.7	−2.8	2246.3

*Performance of deep learning with artificial neural networks optimized with IEL for two predictive targets is shown and compared with performance without IEL using default settings for hyperparameters. Performance metrics for the best-performing model validated on held-out data are shown*.

### IEL Provided Explainable Deep Learning and Ranked Predictors by Importance

Using the feature selection properties of IEL, we were able to provide explainable models for all three algorithms including deep learning with artificial neural networks. This enabled direct comparisons to be made of the most important predictors and their relative importance for experiments using different techniques (Tables 5, 6).

**Table 5:** Optimized predictors of life function and autism.

	**Life function**		**Autism**	
	**Features**	**Imp**	**Features**	**Imp**
Deep learning: classification	Autism traits (ASSQ) Hyperactivity traits (SDQ) Mood symptoms (MFQ) Social skills (SRS) Internalizing traits (SDQ) Social aptitude (SAS) Repetitive behaviors (RBS)	0.26 0.21 0.20 0.20 0.18 0.16 0.16	Non-verbal communication (SCQ) Internalizing traits (SDQ) Repetitive behaviors (RBS) Functional impairment (CIS) Hyperactivity (SDQ) Gets along with school peers Mood symptoms (MFQ)	0.29 0.21 0.16 0.15 0.14 0.14 0,14
Deep learning: regression	Social skills (SRS) Functional impairment (CIS) Internalizing traits (SWAN) Mood symptoms (MFQ)	−71.2 −29.3 −24.5 −21.3	-	-
Decision tree	Social skills (SRS) Visual/language network connectivity	0.71 0.29	Social skills (SRS) Annual household income Takes psychiatric medications Diastolic blood pressure Internalizing traits (SDQ) Mood symptoms (MFQ, P) Newborn problems Mother took medication during pregnancy Bullied by peers	0.41 0.11 0.11 0.08 0.07 0.07 0.05 0.04 0.04
Linear	Social skills (SRS)	−0.92	Internalizing traits (SDQ) Nonverbal communication (SCQ) Functional impairment (CIS)	−0.42 −0.39 −0.14

*For each algorithm type, we show predictors of individual life function and autism and their relative importances (Imp) for the best-performing model (Table 4) after optimization with IEL. Importances are computed as detailed in materials and methods. Names of the specific psychometric assessment (Supplementary Table 1) used as the basis of the predictor are shown in parentheses*.

**Table 6:** Optimized predictors of problem behaviors.

**Problem behaviors**	**Features**	**Imp**
Deep learning: regression	Functional impairment (CIS)	−44.1
	Externalizing traits (SDQ)	−35.1
	Social skils (SRS)	−31.9
	Mother took medication during pregnancy	−3.5

*Predictors of problem behaviors and their relative performance are shown for regression-based prediction for the best-performing model (Table 4) after optimization with IEL. Importances (**Imp**) are computed as detailed in Materials and Methods. Names of the specific psychometric assessment (Supplementary Table 1) used as the basis of the predictor are shown in parentheses*.

### IEL Provided Substantial Performance Improvements vs. Default Hyperparameter Settings

Substantially improved predictive performance was obtained by applying IEL to ML algorithms (Tables 3, 4) when compared with conventional learning using default hyperparameter settings. The performance improvement obtained with IEL was most pronounced in deep learning with artificial neural networks, where we saw 20–35% higher accuracy, precision and recall in classification and 45–70% better *R*^2^ and explained variance in regressions. IEL particularly improved accuracy and recall in decision-tree based learning with XGBoost and the linear ElasticNet models. More modest improvements were seen in precision statistics in these latter two algorithms.

Table 7 shows the differences between IEL-optimized and default hyperparameter settings. In all cases hyperparameters tuned with IEL in our optimized solutions differed from the default values offered by the nested ML algorithms, in many cases substantially. For the ANNs, the optimal learning rate was noticeably higher than the default. In the case of the gradient-boosted decision tree-based solutions, maximum tree depth was slightly higher at 7 vs. a default of 6. We found that IEL added a small value for the node partition threshold (where the default is 0). The most striking difference from default settings came in the L1 penalty (“alpha”), where IEL added a substantial amount of regularization. The default settings in the machine learning algorithms are 0 for XGBoost and 0.15 for L1 and L2 in the linear ElasticNet technique. IEL increased the L1 penalty substantially but preferred a lower L2 penalty than the default.

**Table 7:** Hyperparameter values after tuning with IEL.

**Algorithm type and hyperparameters**	**Optimal hyperparameters**	**Default**
**(a)**		
**Artificial neural network**		
Learning rate	0.003	0.001
Beta 1	0.984	0.900
Beta 2	0.982	0.999
**XGBoost (tree-based)**		
Maximum tree depth	7	6
Node partition threshold (gamma)	3.98^−06^	0
L1 penalty (alpha)	0.46	0
**ElasticNet (linear)**		
L1 penalty	0.49	0.15
L2 penalty	0.08	0.15
**(b)**		
**Artificial neural network**		
Learning rate	0.004	0.001
Beta 1	0.941	0.900
Beta 2	0.941	0.999
**XGBoost (tree-based)**		
Maximum tree depth	7	6
Node partition threshold (gamma)	9.94^−06^	0
L1 penalty (alpha)	0.64	0
**ElasticNet (linear)**		
L1 penalty	0.34	0.15
L2 penalty	0.33	0.15

*Hyperparameter values achieved after tuning with IEL for classification-based prediction are shown for the best-performing model in each algorithm class for a Life function and b Autism*.

### The Number of Generations Required to Optimize Learning Varied Across Experiments

IEL is an adaptive algorithm that learns during optimization over successive generations as it encounters new data on an individualized basis for each machine learning technique and experiment. We found that the number of learning generations required for the fitness function (Bayes Information Criterion) to plateau varied among individual experiments (Table 8).

**Table 8:** Number of generations required for fitness function to plateau during training.

**Experiment**	**Algorithm type**	**Number of generations**
Life function classification	ANN	107
	XGBoost	33
	ElasticNet	130
Autism classification	ANN	30
	XGBoost	133
	ElasticNet	140
Life function regression	ANN	175
Problem behaviors regression	ANN	60

*The number of learning generations required for the Bayes Information Criterion to plateau (Figure 3) during initial training is shown for each algorithm and experiment type*.

## Discussion

Evolutionary learning is a metaheuristic that offers compelling advantages when applied to machine learning as an AI optimizer: it learns adaptively, surveys the search space randomly, is representation independent (e.g., accepts categorical variables) and is intuitive and transparent. Metaheuristic methodologies have been an active area of research for decades and are often inspired by natural, stochastic phenomena like genetic selection, particle swarms (which have been applied to model hyperparameter tuning) or insect colony behavior (Liang et al., 2020). They have made major impacts in providing practical solutions to combinatorial problems in diverse scientific fields (Osman and Laporte, 1996). For example, a conceptually similar problem exists in constructing models to fit experimental observations in biochemistry and thermodynamics: evolutionary algorithms have been applied to overcome local minima problems arising from dependence on an initial user-provided “guess” of the standard non-linear least squares technique (Ingram et al., 2021). Here, we use evolutionary learning in a novel narrow AI application to jointly learn features and hyperparameters and thereby optimize machine learning. The overall aim is to provide practitioners with a principled approach to unconstrained learning problems in discovery computational science, particularly in large and/or high-dimension datasets where many potential predictors are available and approximate hyperparameter ranges unknown. Evolutionary learning has previously been selectively applied to hyperparameter tuning but not to our knowledge to solve the problem of automated feature selection. In particular, we are not aware of AI methods which use evolutionary learning to jointly learn features and hyperparameters. Comparisons to other methods for feature selection or hyperparameter tuning are successively discussed below.

Hyperparameter optimization is of immediate and pragmatic relevance to machine learning practitioners given its impact on model training and performance. It is highly germane in deep learning where hyperparameter “tuning” can be particularly challenging. Besides manual selection, the standard automated approaches are grid search and randomized parameter optimization, both implemented in widely used packages such as scikit-learn (https://scikit-learn.org/stable/modules/grid_search.html). The former is probably the most popular technique, where a grid is constructed of candidate hyperparameters and all combinations of these are exhaustively attempted during training to identify the best-performing set. While this can be computationally manageable if the grid is relatively small, implementation becomes unwieldy if a large number or wide ranges of hyperparameters are under consideration as is the case in unconstrained and/or novel learning problems. Randomized parameter optimization (Bergstra and Bengio, 2012) attempts to reduce such computational demands by assembling hyperparameter sets via sampling of distributions over possible parameter values. Both methods are somewhat “brute force” approaches that are non-adaptive (i.e., do not take advantage of prior learning) and select discrete sets of hyperparameter values, thereby running the risk of limiting the search space and introducing bias. Unless the entire search space is sampled, there is no guarantee of finding a local minimum. More recently, Bayesian techniques such as Spearmint (Snoek, 2012) (https://github.com/HIPS/Spearmint) have gained in popularity and can offer efficiencies in terms of evaluating the chosen objective function, though this benefit tends to degrade as the search dimension increases. Bayesian methods also sample the hyperparameter space to construct a surrogate model, but can be computationally expensive given their sequential nature and/or limited to the optimization of continuous hyperparameters, though newer efforts to parallelize these techniques and allow better scaling have had promising results (Snoek, 2015).

In recent years, the potential for evolutionary learning to address the difficult problem of hyperparameter optimization for deep learning has been recognized. Hyperparameter settings in deep learning have complex effects on model performance that can differ by the type and relative complexity of the learning architecture and the dataset being analyzed (Bruel, 2015). Early studies applied evolutionary algorithms to optimize hyperparameters in shallow ANNs with a single hidden layer and demonstrated superior performance and the promise of these methods (Cantu-Paz and Kamath, 2005; Fiszelew, 2007). More recently, several studies have explored the ability of evolutionary algorithms to optimize hyperparameters for the deeper, more complex ANNs that are increasingly used in scientific research. Young et al. applied an evolutionary algorithm with 500 models per generation over 35 generations and error as the fitness function, classifying color images from the CIFAR-10 dataset (Young, 2015). This technique differs from IEL in that feature selection is not provided and the number of learning generations is fixed at the outset rather than including a principled convergence criterion of a fitness function plateau. Without a dynamic convergence criterion, there is a risk that training may be terminated too early with a smaller number of learning generations, foregoing a stable and optimized minimum. However, this work did demonstrate that fitness improved over successive generations using evolutionary learning. Cui et al. also focused on CIFAR-10, using an evolutionary approach hybridized with a Gaussian-based Bayesian method to optimize both hyperparameters and the number of kernels and layers to improve the classification performance of convolutional neural networks, again using error as the fitness function over a fixed number of 1,000 learning generations (Cui and Bai, 2019). Interestingly, this technique revealed that hyperparameters stabilized at minima long before 1000 generations, illustrating the obverse risk: without convergence criterion, learning can go on too long and become computationally inefficient. Similarly, feature selection was not integrated as it is with IEL. We also note that both methods focus on the CIFAR-10 dataset – a benchmark machine learning problem that functions as a constrained system with known predictive features and approximate hyperparameter ranges.

Our results show that very robust and consistent performance can be achieved in complex, multi-domain data using IEL, particularly utilizing deep learning with ANNs. IEL's performance offers very substantial performance gains over baseline training with default hyperparameter settings in both classification and regression. Including convergence concepts grounded in information theoretic and performance metrics offers a principled way to calibrate and quantify the amount of training required. By performing a variety of experiments in the present study we show how the number of learning generations (and computational effort) can vary widely among experiments from 30 to 175 generations, pointing up the value of using a quantitative convergence horizon as well as an adaptive learning method. These results suggest that empirically selecting a number of iterations such as 35, 50, or 1,000 generations to train a model runs the risk of a sub-optimal solution or computational inefficiency. Illustrative comparisons can be made to quantify the value of IEL's convergence strategy. For example, here we show that tuning 3 ANN hyperparameters with IEL requires training 30,000 to 175,000 models (100 models per 30–175 generations) where the requisite hyperparameters (learning rate, beta 1, beta 1) could assume values over a range size of ~99 (Table 2). To explore the same sized solution space of 99 × 99 × 99 with similar resolution using grid search would require training nearly a million ANNs per experiment, a much larger computational load. Similarly, the substantial difference in tuned hyperparameters after optimization with IEL vs. default algorithms settings may suggest the value of adaptive optimization in achieving robust results. Besides the principled approach offered by IEL to hyperparameter tuning, efficiencies may be garnered thereby within the machine learning processes. For example, we found a faster (higher) learning rate was optimal in deep learning than that suggested in the default parameter, speeding the machine learning. While methods such as grid search can be efficient when a constrained or familiar model system is under consideration with a small number of preselected features and relatively narrow range for hyperparameters to be tuned, IEL offers advantages with more unconstrained and/or novel problems where feature selection is required and “rules of thumb” for hyperparameter settings are unknown. As well, IEL can be used for exploratory analyses where practitioners might explore settings for individual datasets and developing their own “rules of thumb” to constrain the hyperparameter space for any particular dataset, perhaps going on to use grid search or Bayesian techniques once these constrained ranges have been established.

Feature selection is a similarly essential and challenging part of machine learning at scale, particularly in multi-domain and/or high-dimension datasets. In discovery science in such datasets where hundreds or thousands of potential predictors are available, feature selection is a requirement. Generally, models with fewer variables are simpler to train, run and understand and generalize better to unseen data. As with hyperparameter optimization, a mixture of manual and automated approaches has been attempted. Historically, practitioners adopted a “domain-informed” manual approach, selecting predictors from a larger set based on personal heuristics informed by prior research, domain knowledge or hypotheses. Besides the risk of bias, manual feature selection is challenging in hypothesis-free research or when we have insufficient information to make determinations. Exploratory data analysis is often undertaken to narrow the number of predictors. Preliminary preprocessing steps such as thresholding, identifying correlated variables or applying information theoretic metrics may help in “tuning down” the number of potential predictors. However, we are often still left with many potential predictors and moreover ranking predictors by importance may be a useful or explicit experimental aim. In this case, using model-based feature selection is a powerful tool to discover the relative importance of individual predictors and prune those which prove less important. Typically, practitioners needing to quantify the relative importance of predictors have turned to linear models and decision tree algorithms, since these intrinsically provide feature importances. These can be combined with simple recursive feature selection (e.g., https://scikit-learn.org/stable/modules/generated/sklearn.feature_selection.RFE.html) to prune features. However, the number of features to keep and discard must still be manually selected.

Applying IEL to feature selection allows the best-performing features to be learned and underperforming features recursively pruned in an adaptive, principled and integrated manner. This application of evolutionary algorithms has been of interest for some time, and in fact showed much earlier promise in outperforming other methods with large, noisy datasets (Vafaie, 1994; Oh et al., 2004). More recently, evolutionary algorithms have continued to perform well in selecting features in heterogenous biomedical datasets, for example electroencephalographic signals (Saibene, 2021), heart disease (Abdollahi, 2021) and ovarian cancer. In IEL, we extend the powerful ability of evolutionary algorithms to learn adaptively to the problem of feature selection by calibrating feature fitness with an information theoretic metric and combining feature selection with hyperparameter tuning. This integrated approach allows IEL to rank features by their importance to model predictions and prune features in a principled and adaptive manner based on an objective fitness function. Feature importances to prediction are returned, even during deep learning. As a result, we avoid overfitting and retain transparency with respect to which features are driving predictions across all algorithmic types, enabling direct comparison of experimental outcomes among machine learning techniques including deep learning with ANNs.

Overall, our results show that optimization with IEL provides excellent, consistent performance, in particular using deep learning. IEL's ability to successively prune features and select the best-performing predictors over generations of learning avoids over-fitting and provides robust generalization to unseen validation data. Substantial performance gains accrue from applying IEL over conventional training and testing with default hyperparameters. We demonstrate that this is the case for multiple ML algorithm types, but it is particularly apparent in deep learning with artificial neural networks, where 20–70% improvements across performance statistics were obtained in both classification and regression. Applied to deep learning with ANNs, IEL is able to achieve ≥95% accuracy, specificity and sensitivity in classification in complex bio-behavioral data. Our primary motivation in constructing IEL was to offer researchers analyzing complex, multi-domain biological or bio-behavioral data the ability to not only optimize models but also successfully reduce the feature space in a principled manner and preserve feature explainability across a variety of AI/ML techniques where the problem at hand is novel and/or unconstrained. IEL opens up the potential to attack bio/behavioral “big data” for discovery science with techniques such as deep learning in an efficient manner. We do note that given the adaptive, evolutionary learning process embodied by IEL, substantially increased training times are required vs. conventional training. In the simplest case, training the linear models with IEL takes 5–6 min vs. seconds. This penalty increases for decision-trees and is most severe with deep learning with artificial neural networks. In the present experiments, training the former took 7–9 h and the latter up to 100 h. While evolutionary techniques can be time-consumptive depending on the characteristics of the dataset and experiment, we believe that for multi-domain, complex data and problems IEL compensates the practitioner with valuable additional functionality and high-quality robust solutions that generalize well. In the present study, we implemented IEL on a single GPU to democratize our results, since many practitioners (particularly early-stage investigators) may not have access to larger scale compute resources. Future directions will likely include a parallelized implementation of IEL for faster computation in larger datasets and an evolutionary method including fitness convergence criteria that focuses only on hyperparameter tuning to enable instructive comparisons with techniques such as grid search and Bayesian optimization.

## Data Availability Statement

The dataset analyzed for this study can be found in the Healthy Brain Network Data Portal (http://fcon_1000.projects.nitrc.org/indi/cmi_healthy_brain_network/).

## Author Contributions

NL designed the experiments, coded the algorithms, performed the computations and experiments, and wrote the paper. JK designed the experiments and algorithms and contributed to writing the paper. MR contributed to algorithm coding. All authors contributed to the article and approved the submitted version.

## Conflict of Interest

The authors declare that the research was conducted in the absence of any commercial or financial relationships that could be construed as a potential conflict of interest.

## Publisher's Note

All claims expressed in this article are solely those of the authors and do not necessarily represent those of their affiliated organizations, or those of the publisher, the editors and the reviewers. Any product that may be evaluated in this article, or claim that may be made by its manufacturer, is not guaranteed or endorsed by the publisher.
