# Supplementary material for: Integrated Evolutionary Learning: An Artificial Intelligence Approach to Joint Learning of Features and Hyperparameters for Optimized, Explainable Machine Learning
Source: Front Artif Intell. 2022 Apr 5;5:832530. doi: 10.3389/frai.2022.832530 (PMC9038845; doi:10.3389/frai.2022.832530)
Supplement: Supplementary file 1 [file Table_1.DOCX]

**Supplementary Table 1**

List of assessments from which feature predictors were drawn with brief description of their contents.

| **Measure** | **Description** | **Detail** |
| --- | --- | --- |
| ESPAD | Drug and alcohol screening | Ever used marijuana |
| ESPAD | Drug and alcohol screening | Computed composite score of all substances |
| PhenX Neighborhood | Feelings about neighborhood safety |  |
| Age | Age at assessment |  |
| Sex | Sex at birth |  |
| PPS | Interview based puberty rating scale |  |
| FSQ | Financial support questionnaire | Type of health insurance |
| FSQ | Financial support questionnaire | Parent currently employed |
| FSQ | Financial support questionnaire | Annual household income |
| MRI site | Site at which MRI performed |  |
| PBQ | Pregnancy and birth questionnaire | Mother ever smoked |
|  | Pregnancy and birth questionnaire | Mother ever smoked during pregnancy |
|  | Pregnancy and birth questionnaire | Mother had medical problem requiring treatment |
|  | Pregnancy and birth questionnaire | Mother took prescribed medication during pregnancy |
|  | Pregnancy and birth questionnaire | Mother took illicit drugs during pregnancy |
|  | Pregnancy and birth questionnaire | Mother consumed alcohol during pregnancy |
|  | Pregnancy and birth questionnaire | Child birth weight |
| Pre-Interview Demographics | Race | African American |
| Pre-Interview Demographics | Race | Caucasian |
| Pre-Interview Demographics | Race | Hispanic |
| Pre-Interview Demographics | Race | Other |
| Pre-Interview Developmental History |  | Birth complications |
| Pre-Interview Developmental History |  | Infant temperament |
| Pre-Interview Developmental History |  | Normal development |
| Pre-Interview Developmental History |  | Growth concerns |
| Pre-Interview Developmental History |  | Newborn problems |
| Pre-Interview Developmental History |  | History of prematurity |
| Pre-Interview Developmental History |  | Mother’s age at birth |
| Pre-Interview Developmental History |  | Skill loss prior to age 6 |
| Pre-Interview Developmental History: | Pregnancy symptoms | Maternal emotional issues |
| Pre-Interview Developmental History: | Pregnancy symptoms | Threatened miscarriage |
| Pre-Interview Developmental History: | Pregnancy symptoms | Family stress |
| Pre-Interview Developmental History: | Pregnancy symptoms | Flu or other virus |
| Pre-Interview Educational History |  | Early Intervention |
| Pre-Interview Educational History |  | Individualized Education Plan |
| Pre-Interview Educational History |  | Neuropsychological Testing |
| Pre-Interview Educational History |  | After school teams |
| Pre-Interview Educational History |  | Has a best friend |
| Pre-Interview Educational History |  | Family is religious |
| Pre-Interview Educational History |  | Learning disability |
| Pre-Interview Educational History |  | Gets along with kids at school |
| Pre-Interview Educational History |  | Bullied |
| Pre-Interview Educational History |  | Get along with kids in neighborhood |
| Pre-Interview Educational History |  | Number of friends |
| Pre-Interview Educational History |  | Discipline problems |
| Pre-Interview Treatment History |  | History of emergency room visits |
| Pre-Interview Treatment History |  | Firearms in home |
| Pre-Interview Treatment History |  | History of head injuries |
| Pre-Interview Treatment History |  | History of health (medical) problems |
| Pre-Interview Treatment History |  | Currently takes psychiatric medications |
| Pre-Interview Treatment History |  | Has taken psychiatric medications in the past |
| Pre-Interview Treatment History |  | History of (physical) trauma |
| Pre-Interview Treatment History |  | Homicidal thoughts or actions |
| Pre-Interview Treatment History |  | Has been hospitalized |
| CSSRS | Severity of suicidal thoughts and behaviors | Ever wished dead or to not wake up |
| IAT | Behaviors associated with internet use | Total score |
| PCIAT | Compulsive use of internet | Total score |
| Connors | ADHD rating scale | Subscale scores |
| Connors | ADHD rating scale | Parent negative impression of child |
| Connors | ADHD rating scale | Parent positive impression of child |
| CCSC | Child’s coping strategies | Computed composite score |
| CPIC | Child’s perception of parental conflict |  |
| PSI | Stress in parent-child system |  |
| ICU | Callous and unemotional traits |  |
| SCQ | Nonverbal social communication |  |
| SDQ | Behavioral screening questionnaire | Hyperactivity traits |
| SDQ | Behavioral screening questionnaire | Internalizing traits |
| SDQ | Behavioral screening questionnaire | Prosocial traits |
| SDQ | Behavioral screening questionnaire | Externalizing traits |
| CTOPP | Phonological processing |  |
| Pegboard | Test of manipulative dexterity | Dominant hand |
| Pegboard | Test of manipulative dexterity | Nondominant hand |
| Physical assessment |  | Diastolic blood pressure |
| Physical assessment |  | Body Mass Index |
| SRS | Autism-oriented measure of interpersonal, communication skills and repetitive behaviors | Total score |
| SWAN | Parent report of psychopathology | Hyperactivity scale |
| SWAN | Parent report of psychopathology | Internalizing trait scale |
| TOWRE | Pronunciation of printed words |  |
| Full Scale Intelligence Quotient | Composite measure of general intelligence |  |
| WIAT | Achievement skills assessment | Listening comprehension |
| WIAT | Achievement skills assessment | Numerical operations |
| WIAT | Achievement skills assessment | Reading comprehension |
| FGC | Physical fitness test |  |
| SAS | Social aptitude |  |
| NLES | Family, community and school stressors and their effects |  |
| BIA | Bioelectric impedance analysis | Daily energy expenditure |
| CIS | Function in relationships, psychopathology, school and leisure |  |
| MFQ | Frequency of mood and feeling states |  |
| NIH Toolbox | Cognitive function test | Card sort score |
| NIH Toolbox | Cognitive function test | Flanker score |
| NIH Toolbox | Cognitive function test | List pattern score |
| NIH Toolbox | Cognitive function test | Pattern recognition score |
| Temporal Discounting | Delay discounting task | Run 1 |
| Temporal Discounting | Delay discounting task | Run 2 |
| PANAS | Mood scale | Negative Affect score |
| PANAS | Mood scale | Positive Affect score |
| ASSQ | Symptom checklist for autism |  |
| Barratt | Measure of social status | Total educational score |
| Barratt | Measure of social status | Total occupational score |
| DTS | Distress tolerance |  |
| RBS | Breadth of repetitive behaviors |  |
| Edinburgh Handedness Scale | Dominance of right or left hand |  |
| GFTA | Spontaneous and imitative articulation of consonant sounds | Sounds in words score |
| APQ | Parenting style and behaviors |  |
| CGAS | Global assessment of function |  |
