# Supplementary material for: Integrated Evolutionary Learning: An Artificial Intelligence Approach to Joint Learning of Features and Hyperparameters for Optimized, Explainable Machine Learning
Source: Front Artif Intell. 2022 Apr 5;5:832530. doi: 10.3389/frai.2022.832530 (PMC9038845; doi:10.3389/frai.2022.832530)
Supplement: Supplementary file 2 [file Table_2.DOCX]

**Supplementary Table 2: Pseudocode for the IEL algorithm**

Inputs: IEL (evolutionary algorithm) parameters; feature set; prediction targets, generation limit

**for** target in prediction targets do:

if desired, rebalance sampling data (we used SMOTEENN)

set hyperparameter values and numbers of features with random values in desired ranges

Create empty fitness_list, feature_importances_list

#run n=100 models with all permutations in initial generation of evolutionary learning

**for** each matrix of hyperparameter settings and feature sets in initial generation do:

sample X

Create empty fitness_scores, feature_importance_scores

compute k for cross-validation

**for** each fold in cross-validation do:

train model on fold

store model’s scores in fitness_scores, feature_importance_scores

compute and store mean(fitness_scores) in fitness_list

compute and store mean(feature_importance_scores) in feature_importances_list

rank models by performance and store in ranked_fitness

create queue to store top 3 performing models from each generation and initialize with first 3

Create empty lists for best fitness function, feature sets and hyperparameter settings

#Continue genetic algorithm for second to final learning generations

initialize second learning generation, g, and convergence condition = False

**while** g < total number of desired learning generations and converged == False do:

#mating of parent models

set parents = select top 40 models

set children = generate 20 children by crossover at pivot point (hyperparameters, feature sets) from parents

#mutation of parent models

set mutations = select next 20 best-performing models

set mutation_children = change value of hyperparameter by specified amount

# establish new generation of child models

set new_children = children + mutation_children

initialize 60 new random_children from distributions

set new population_arrays = new_children + random_children

initialize score_lists based on various metrics

**for** each matrix of hyperparameter settings and feature sets in population_arrays do

sample X

initialize empty metric lists (fitness_list, statistical metrics of choice)

compute k for cross-validation

**for** each fold in dataset do

train model on fold

store model’s scores in metric lists

compute and store mean(metrics) in mean metric lists

set ranked_fitness = fitness_list

order ranked_fitness based on fitness_score and add top 3 with their hyperparameters to best_lists

add top fitness models to queue
 #determine convergence or continuance of learning

**if** length(queue) < minimum size of queue set in IEL parameters

increment g by 1 and continue learning

**else**

**if** convergence condition not met

delete first column from queue

increment g by 1 and continue learning

**else** stop learning
